# Spinal Pseudoaneurysms Mimicking an Osteogenic Tumor: A Case Report

**DOI:** 10.5435/JAAOSGlobal-D-19-00156

**Published:** 2020-05-01

**Authors:** Mohammed AlSalman, Sami Aleissa, Ali Alhandi, Raghad AlSayari, Nawaf Alamri, Fahad Alhelal, Majed Abalhkail, Faisal Konbaz

**Affiliations:** From the National Guard Health Affairs, King Abdulaziz Medical City, Riyadh, Saudi Arabia.

## Abstract

**Aim:**

This study presents a rare case of pseudoaneurysm mimicking a tumor on the back, with no history of fever, trauma, or surgical intervention. In which no identifiable symptoms or warning signs were present. This pseudoaneurysm arises from intercostal arteries and segmental arteries. Both of which, to the limit of our knowledge, have not been discussed before.

**Method:**

This study was done using chart and literature review. We present a case of a 46-year-old man with a known case of left-sided intracranial hemorrhage due to hypertension and an old cerebrovascular accident. The patient has a positive history of hypertension, which could have attributed to the pseudoaneurysm; however, he has no history of vascular disease otherwise. The patient reports of back swelling and intermittent back pain for the past 3 years. On MRI, the mass showed a pulsating pattern around it. It also showed a layering effect because of different wall thicknesses and enhanced patterns, and the enhancement ratio was increased. In addition, it showed flow artifacts with T1 hyperintense areas because of associated thrombus and blood products. These changes noted on the MRI prompted the team to do a color Doppler study to confirm the presence of an aneurysm and if present, to do a CT angiography. The color Doppler showed a turbulent flow, that is, there was a bidirectional pulsatile flow which further confirms the presence of a pseudoaneurysm. Spine CT with contrast showed a right paraspinal lesion at the T9-T11 level. It had contrast enhancement and flow inside, consistent with a partially thrombosed aneurysm. The CT also showed evidence of bone remodeling in the adjacent thoracic vertebrae. The patient opted for spinal vascular emobilization and vascular sheath removal. The right and left intercostal arteries were selected at the level of left and right T4, left T8, bilateral T9, and bilateral T10.

**Conclusion:**

Differentiating between pseudoaneurysms and osteogenic tumors is essential to target later investigations accordingly. In addition, if pseudoaneurysms are left untreated, they could cause bony erosions of the vertebra, which lead to compression fractures. They can further compress the adjacent neurovasculature, which worsens the morbidity.

Aneurysms are localized swellings in the walls of an artery, usually because of a weak spot. They can be true, with all three vessel walls, or pseudoaneurysms, where an opening in one of the vessel walls allows leakage of blood through its wall.^1^ This leakage is then contained by scar tissue or the adventitia itself.^1^ Pseudoaneurysms can mimic bone tumors or soft-tissue sarcomas.^2^ They also occur more commonly in patients of relatively the same age of those patients with sarcoma and tumor, that is, between the ages of 20 and 60 years. The mechanism and causation of pseudoaneurysm is not exactly known. However, it has been proposed that they result from a gradual growth that occurs because of constant arterial pressure and the development of a reactive fibrous capsule.^3^ Moreover, they usually occur after a penetrating injury, blunt trauma, or an endovascular procedure.

Differentiating between pseudoaneurysms and osteogenic tumors clinically is challenging because they share similar signs and symptoms. They also share a similar presentation on a CT scan because they both appear as a lesion or a mass. Moreover, CT scans show bony erosions that further support the overlap between them and osteogenic tumors (Figure 1). This overlap might lead to the medical decision to further investigate the mass and even intervene by aspiration or obtaining a tissue biopsy. This will eventually lead to morbidity and sometimes even mortality. Thus, this medical decision relies heavily on a multidisciplinary team approach.^3^

**Figure 1:**
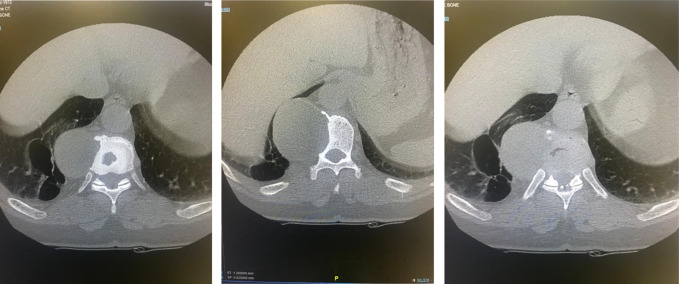
Radiograph showing the axial view of the bony changes caused by the pseudoaneurysm.

Pseudoaneurysms represent a pulsating encapsulated hematoma in contact with the lumen of a ruptured vessel. They usually occur after a disruption in the arterial wall continuity, which leads to blood dissecting into the surrounding soft tissues without including the arterial wall in the aneurismal sac.^4^ Pseudoaneurysms in the spine can occur because of an iatrogenic, traumatic, neoplastic, infective, vasculitic, or inflammatory etiology. They can also occur in several sites, such as knees and elbows.^4^ The site of the pseudoaneurysm usually suggests the underlying cause (eg, iatrogenic and neoplastic pseudoaneurysms, although rare, occur in the back).

This study presents a rare case of pseudoaneurysm mimicking a spinal tumor, with no history of fever, trauma, or surgical intervention. This pseudoaneurysm has risen idiopathically from intercostal arteries and segmental arteries, both of which, to the limit of our knowledge, have not been described before.

## Case Presentation

We present a case of a 46-year-old man living in a remote rural area with a history of an old left-sided intracranial hemorrhage. The patient had lingering neurologic deficit from his previous stroke and presented with a report of intermittent back pain for the past 3 years. The pain first started as pressure-like in nature, 2 of 10 in intensity, not radiating, and occurring once every 2 to 4 weeks. Two weeks before presenting at our institution, the patient experienced gradual increase in his pain symptoms, the pain was continuous and pressure-like in nature (6 to 7 of 10), and radiating to the upper and lower spine without limb involvement; however, it was controlled with simple analgesia. One week later, the patient sought medical treatment in an outside facility where tramadol was needed to control the pain (dosages unknown). The patient denied any limb weakness, radicular pain or numbness, and urinary or stool incontinence at the time. The patient was referred to our institution's spine unit for assessment. His referral included an MRI (without contrast) and a medical report suggesting an osteogenic tumor.

On examination, the patient was vitally stable. He had a normal gait; however, the patient could not walk in a tandem gait or on tiptoes/heels alone. It was noted that the pain increases with axial rotation of the abdomen and lateral bending. Bilateral lower limb examination showed normal motor function and reflexes. Sensory examination revealed hypoesthesia to pinprick and intact vibration sense in the left lower until midthigh, all of which were present since his previous brain hemorrhage. The examination was otherwise normal.

The plan was to control the pain, monitor distal neurovascular status, rule out brucellosis by a brucella titer, and repeat erythrocytes sedimentation rate and C-reactive protein tests, in addition to doing an acid-fast bacilli test and culture for tuberculosis.

**Figure 2:**
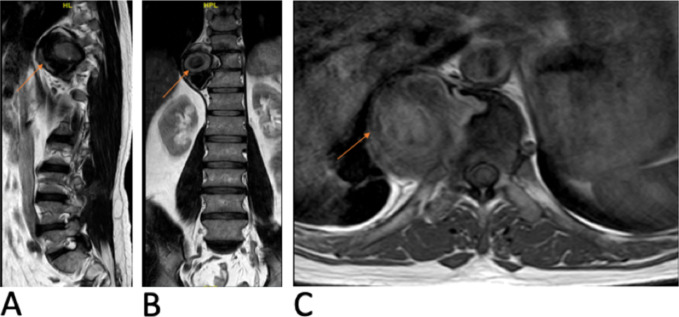
Radiograph of the lumbar and thoracic spine MRI without contrast. **A** and **B**, Sagittal and coronal T-2 weighted image showing a right paraspinal lesion with heterogeneous signal and concentric layering. **C**, Axial T1-weighted imaging showing pulsation artifact.

However, a secondary review of his MRI by a senior musculoskeletal radiologist leaned toward a possible vascular etiology (T2-weighted sequences: the mass showed heterogeneous T2 signal with concentric layering and central signal void. T1-weighted sequence: the mass showed mild heterogeneity with intermediate to high T1 signal with pulsation artifact, Figure 2). The findings were suggestive of a partially thrombosed pseudoaneurysm. Contrast studies were needed, but the decision was quickly forgone after further assessment because the patient had elevated creatinine (171 umol/L) and low estimated glomerular filtration rate (40 mL/min/1.73 m^2^).

**Figure 3:**
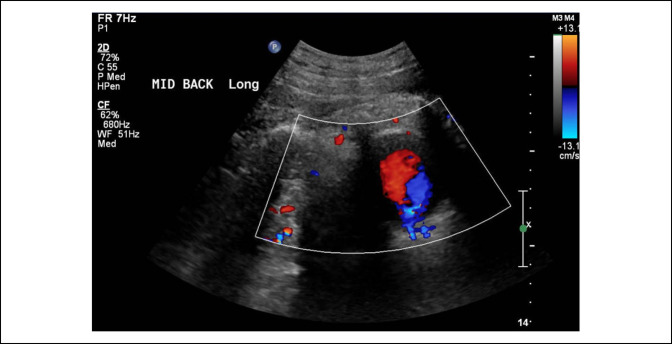
Radiograph showing color Doppler US of the back showing a bidirectional swirling flow consistent with the pseudoaneurysm.

A color Doppler ultrasonography (US) study (Figure 3) was recommended instead to confirm the presence of a pseudoaneurysm and if present, to proceed with CT angiography. The color Doppler US showed a bidirectional and pulsatile turbulent flow, further confirming the presence of a pseudoaneurysm.

**Figure 4:**
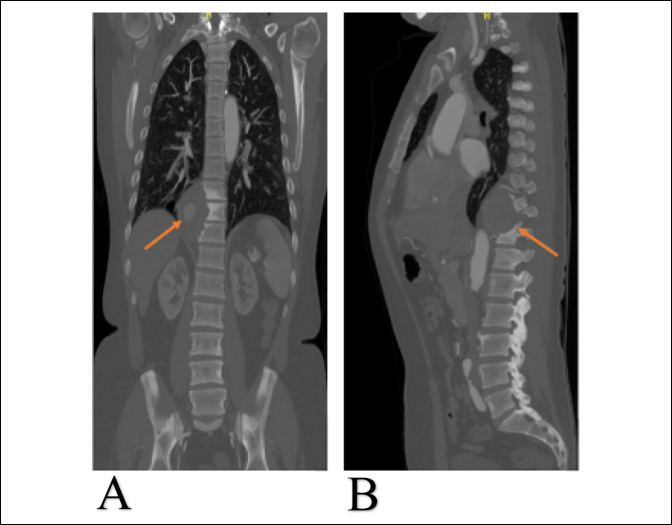
Radiograph showing the CT angiogram of the spine. **A**, Coronal view of pseudoaneurysm noted with arrows showing partial contrast enhancement inside the pseudoaneurysm. **B**, sagittal view of the pseudoaneurysm appearing as a mass spanning T-9 to T11.

Nephrology consultation was made because the patient was expected to undergo multiple contrast-enhanced studies and/or treatment modalities. After proper optimization and clearance, we proceeded with CT angiogram that showed a right paraspinal mass at the T9-T11 level (Figure 4). It had contrast enhancement and flow inside, consistent with partially thrombosed pseudoaneurysm. The lesion was thought to be arising from the right T10 segmental or intercostal artery. Evidence of bone remodeling in the adjacent thoracic vertebrae was also noted (Figure 1).

**Figure 5:**
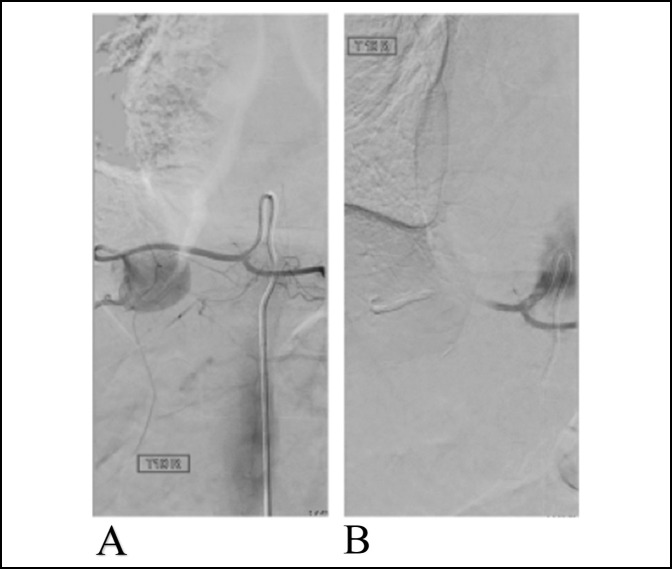
Radiograph of the arterial angiogram of the embolization procedure. **A**, Photograph showing filling of the pseudoaneurysm with contrast. **B**, Postembolization with ONYX 18 with no flow into the pseudoaneurysm.

The procedure was discussed, along with its indication, alternatives, and potential complications, and informed consent was obtained. Using a standard technique, right femoral artery access was obtained. An aortic angiogram was performed, which showed the posterior intercostal arteries at the levels of T8, T9, and T10. A large aneurysm was noted, arising from the midpart of the right T10 segmental artery with a narrow neck, pointing toward the spinal column with an estimated sac of 3 to 4 cm. A tributary to the anterior spinal artery coming from the left T10 segmental artery was also noted. Embolization of the pseudoaneurysm was performed by using Onyx 18 (ONYX liquid embolic system, Medtronic) (Figure 5). Postembolization contrast injection showed no refilling of the pseudoaneurysm.

Postprocedure, the patient was stable and discharged the next day. His pain completely resolved postembolization. Patient was pain free due to the pressure relief that was pressing on his vertebra in association with the pain medications he was discharged on; the patient was discharged on tramadol, acetaminophen, omeprazole, and nifedipine. He was seen in clinic 14 days later with no new symptoms or reports. Follow-up imaging was planned for his 6-week visit, but he was lost to follow up.

## Discussion

Pseudoaneurysms occurring in the spine are thought to be a rare event but more reports are being published recently describing multiple etiologies. Iatrogenic and posttraumatic causes seem to be the more common types reported in the literature.^5,6,7,8,9,10,11,12^ Liu et al recently published a literature review of iatrogenic vascular injuries postspinal surgery. They included 20 case reports totaling 26 patients, of which, 12 patients sustained a pseudoaneurysm during their clinical course. Cappuzzo et al recently published a case report with a literature review pertaining to a delayed traumatic case resulting in pseudoaneurysms in the spine. In their report, they discuss six studies with 47 patients in total. Most of the cases were because of penetrating trauma (60%).^12,13,14,15,16^ There are several other reports in the literature describing posttraumatic spinal pseudoaneurysms with most patients manifesting their symptoms within 1 year.^5,6,17,18,19,20^

Cases of pseudoaneurysms mimicking tumors in their presentation remain exceedingly rare with a missed diagnosis presenting a possible devastating outcome.^3,4^ We found two cases in the literature where a pseudoaneurysm was initially thought to be a soft-tissue tumor using imaging studies. Both case reports discussed a pseudoaneurysm occurring in the distal femur. Bregowda et al presented two patients with a rather short clinical history of pain and swelling (6 and 3 months). Both cases in the report were initially diagnosed as osteogenic sarcoma of the femur. Both cases underwent a comprehensive multidisciplinary review, and a vascular etiology was confirmed before any interventions.^3^ Albert et al paint a similar picture with their report, initially pursing a diagnosis of soft-tissue sarcoma because of MRI findings. However, contrary to the previous report, the patient was eventually taken for an open soft-tissue biopsy which was complicated by a massive hemorrhage. A repair was ultimately done during the same surgery, and the patient did not suffer any lasting consequences. No cases were found in the literature describing a similar presentation in the spine. However, the reports discussed above offer a cautionary reminder of the possible detrimental outcomes of misdiagnosing pseudoaneurysms.

The symptoms noted in the patient described in this report were initially thought to be due to a tumor or an infectious process. Contrast imaging was not preferred because of his marginal kidney function initially. However, Doppler US was the key diagnostic modality that swayed the diagnostic process in the right direction. His history of vascular events, although unclear because of a lack of proper documentation, was also a factor that assisted in pursuing the correct diagnosis. Unfortunately, our patient was lost to follow up after his initial visit to the clinic; however, his pain and swelling resolved on day one postprocedure with no neurologic deficits.

This report, similar to others in the literature, further emphasizes the difficulty in diagnosing pseudoaneurysms and the need for a multidisciplinary approach in such cases. A vascular cause of spinal masses must be ruled out if imaging is not able to diagnose with certainty because of its high morbidity and possible mortality.
